# Impartiality and infectious disease: Prioritizing individuals versus the collective in antibiotic prescription

**DOI:** 10.1080/23294515.2019.1576799

**Published:** 2019-03-25

**Authors:** Bernadine Dao, Thomas Douglas, Alberto Giubilini, Julian Savulescu, Michael Selgelid, Nadira S. Faber

**Affiliations:** a Faculty of Medicine, Nursing and Health Sciences, Monash University, Melbourne, Victoria, Australia;; b Oxford Uehiro Centre for Practical Ethics, Faculty of Philosophy, University of Oxford, Oxford, United Kingdom;; c Oxford Martin School and Wellcome Centre for Ethics and Humanities, University of OxfordOxford, United Kingdom;; d Monash Bioethics Centre, Monash University, Clayton, Victoria, Australia;; e Department of Experimental Psychology, University of Oxford, Oxford, United Kingdom;

**Keywords:** Drug resistance, microbial, drug resistance, bacterial, antibacterial agents, patient preference, medical overuse, ethics

## Abstract

**Background:** Antimicrobial resistance (AMR) is a global public health disaster driven largely by antibiotic use in human health care. Doctors considering whether to prescribe antibiotics face an ethical conflict between upholding individual patient health and advancing public health aims. Existing literature mainly examines whether patients awaiting consultations desire or expect to receive antibiotic prescriptions, but does not report views of the wider public regarding conditions under which doctors should prescribe antibiotics. It also does not explore the ethical significance of public views or their sensitivity to awareness of AMR risks or the standpoint (self-interested or impartial) taken by participants. **Methods:** An online survey was conducted with a sample of the U.S. public (*n* = 158). Participants were asked to indicate what relative priority should be given to individual patients and society-at-large from various standpoints and in various contexts, including antibiotic prescription. **Results:** Of the participants, 50.3% thought that doctors should generally prioritize individual patients over society, whereas 32.0% prioritized society over individual patients. When asked in the context of AMR, 39.2% prioritized individuals whereas 45.5% prioritized society. Participants were significantly less willing to prioritize society over individuals when they themselves were the patient, both in general (*p* = .001) and in relation to AMR specifically (*p* = .006). **Conclusions:** Participants’ attitudes were more oriented to society and sensitive to collective responsibility when informed about the social costs of antibiotic use and when considered from a third-person rather than first-person perspective. That is, as participants came closer to taking the perspective of an informed and impartial “ideal observer,” their support for prioritizing society increased. Our findings suggest that, insofar as antibiotic policies and practices should be informed by attitudes that are impartial and well-informed, there is significant support for prioritizing society.

## Introduction

Antimicrobial resistance (AMR) is a global public health disaster with significant human and economic costs. Yearly estimates of the human lives lost to resistant infections have reached 700,000 people globally (O'Neill 2016) and are expected to exceed 10 million lives per year by 2050 if no actions are taken against AMR (O'Neill 2016). Patients with resistant infections are more likely to develop complications, and are up to three times more likely to die than patients with nonresistant infections (Cecchini, Langer, and Slawomirski 2015). In the United States alone, AMR accounts for 20 billion USD in excess health service costs annually (Smith and Coast 2013).

Antibiotic use in human health care is a major driver of AMR, and an estimated 30% of all outpatient antibiotic prescriptions in the United States from 2010 to 2011 were found to be inappropriate (Fleming-Dutra et al. 2016). Formulary restriction (prohibiting use of particular antibiotics) and preauthorization requirements have proven effective in reducing antibiotic prescriptions and resistance rates in inpatient settings (Davey et al. 2013; Kaki et al. 2011; Quale et al. 1996). However, less coercive measures—such as physician education and clinical guidelines—have been less successful (Davey et al. 2013). This may be partly attributable to the ethical conflict that doctors often face between acting in the best interests of their patients and acting in the best interest of society-at-large when considering whether to prescribe an antibiotic. There is evidence that doctors frequently prioritize individual patient health over public health when deciding whether to prescribe antibiotics—even when doing so contravenes clinical guidelines (Metlay et al. 2002). Many factors contribute to this tendency, but doctors’ perceptions of patient expectations have been found to strongly correlate with unnecessary antibiotic prescriptions (Britten and Ukoumunne 1997; Cockburn and Pit 1997; Coenen et al. 2006; Lado et al. 2008; Macfarlane et al. 1997; Karras et al. 2003).

Although previous studies have found high rates of patient-reported expectations for antibiotics prior to their consultation (Britten and Ukoumunne 1997; Webb and Lloyd 1994; Stivers et al. 2003), it has also been found that doctors tend to overestimate these patient expectations, leading to antibiotic prescriptions that are neither clinically indicated nor expected by their patients (Lado et al. 2008; Lam, Catarivas, and Lauder 1995; Macfarlane et al. 1997). This suggests that doctors may harbor misplaced assumptions about public expectations for antibiotics. Moreover, even when patients do expect antibiotics, catering to these preferences may be incompatible with advancing patient and/or public health (Gonzales et al. 2001).

Nevertheless, compelling arguments have been made for giving public views some role in shaping health care. Some hold that involving citizens in determining health care goals has intrinsic value and is an integral component of democracy (Council of Europe 2000; Department of Health [UK] 1999; Health Canada 2000; World Health Organization 1978; Kim et al. 2009; Wait and Nolte 2006). Others assert that public involvement in health policymaking can reaffirm consumer rights to information, access, choice, and redress (Wait and Nolte 2006). Public engagement in health policymaking may also result in more resilient policies through increasing public support (Bruni et al. 2008; World Health Organization 2007; Charles and DeMaio 1993; Williamson 2014; British Medical Association 2015). Finally, it might be argued that public attitudes have an evidential role in determining the ethical acceptability of a health care policy or practice, for example, because public attitudes can be expected to converge on, or distribute around, the most defensible ethical position (Surowiecki 2004); because it is rational to defer somewhat to one’s peers when faced with difficult moral quandaries (Nguyen 2010, Rowland 2017); or because health care policy should reflect the values of citizens (Kitcher 2001).

We suggest that insofar as public attitudes are used as evidence for ethical acceptability, it is impartial and informed public views that should be sought. This is because impartiality is central to ethics, and ethical judgments grounded on mistaken information are unjustified. Public views can be considered sufficiently impartial and informed when members of the public who are called to express their views become (imperfect versions of) “ideal observers.”

To elucidate this expression, we need to invoke the philosophical tradition of the “ideal observer” in moral theory. According to ideal observer views, what people ought to do when faced with an ethical quandary is either determined or evidenced by what people would do, or would approve of, if they were in ideal circumstances for rational deliberation (e.g., well-informed and free of distractions) and regarding the issue from an impartial point of view (i.e., with an equal consideration for the preferences and the welfare of all the parties involved) (Hare 1981). For example, in one influential version of the ideal observer theory, one ought to do what could be endorsed by someone who is (1) omniscient about facts, (2) omnipercipient, (3) disinterested, (4) dispassionate, (5) consistent, and (6) normal in all other respects (Brandt 1955, 47; Firth 1952, 31). Of course, no human being can ever satisfy these conditions (Harrison 1956, 256). The theory is intended to specify an ideal. However, we can approximate the conditions to a greater or lesser degree depending, for example, on how well-informed and impartial we are. To the extent that we value democratic processes of policy making for epistemic virtues—that is, because they tend to result in more justified policies—it would be desirable that the public’s approach to antibiotic stewardship was informed by an ethical, rather than a merely self-interested, perspective.

Unfortunately, published studies of public attitudes toward doctors’ antibiotic-prescribing practices mainly encouraged participants to view the issue from a self-interested rather than impartial perspective by seeking attitudes towards their own doctors’ prescribing practices and by studying patients soon to have clinical consultations rather than members of the wider public. Participants were also not informed of the social costs of AMR. Alternatively, previous studies asked the public about possible strategies to avoid misuse of antibiotics (Degeling et al. 2018), but not whether it would be acceptable to prioritize society’s interest in the preservation of antibiotic effectiveness over the individual’s interest in using antibiotics to treat certain infections.

Our study aims to identify the attitudes that would be held by members of the public were they to take a better informed and more impartial perspective—that is, a perspective that comes closer to that of an ideal observer—in cases of conflict between the individual’s and society’s interest.

## Methods

### Survey

Our survey (see appendix) investigated participants’ opinions on whether medical decisions should favor an individual patient’s health or collective well-being, in the case of both general health and AMR in particular. Participants were given short scenarios that described a potential conflict between an individual patient’s health and collective well-being in society and were asked, on a 7-point Likert scale, whether “always the individual” (value 1) or “always society as a whole” (value 7) should be prioritized in the given scenario. Scenarios covered hypothetical decisions in two contexts: a generic, not-further-specified doctor’s decision concerning a generic not-further-specified patient; and the participant’s own doctor’s decision concerning the participant. Both contexts were given for the general case and for the case of AMR, giving a total of four scenarios, which were presented to participants in random order (see supplementary materials for all four scenarios). In addition, we collected demographic information (e.g., age, gender, ethnicity, socioeconomic status), and participants completed the Oxford Utilitarianism Scale (OUS) (Kahane et al. 2018), a validated scale measuring people’s individual tendency to endorse utilitarian views, that is, views according to which morality requires one to always maximize expected utility impartially. This was included as some ideal observer theorists have argued that an “ideal observer” would necessarily be utilitarian (Hare 1981). The OUS contains two (highly intercorrelated) subscales, one indicating people’s endorsement of impartial beneficence (OUS-IB) and the second their willingness to accept instrumental harm (OUS-IH) for the greater good. Our study was reviewed and granted ethical approval from the University of Oxford’s Interdivisional Research Ethics Committee.

### Recruitment

Amazon MTurk was used to conduct the survey. Mturk is an online marketplace that allows people to take part in research online, for a small payment (usually in accordance with U.S. minimum wage). MTurk participant samples have been shown to be more representative of the general population than college samples and standard Internet samples often used in research, and MTurk yields high-quality data that have been shown to meet or sometimes exceed psychometric standards used in published research (Buhrmester, Kwang, and Gosling 2011). We aimed to recruit a minimum of 150 participants.

## Results

One hundred and sixty-three U.S.-American participants took part in our survey online via Amazon MTurk. Five participants were excluded from data analysis because they failed attention checks or did not complete the survey, leaving a total *N* of 158. We analyzed the data using SPSS software, checking for differences depending both on context (generic doctor vs. own doctor) and on case (general health case vs. AMR).

### Demographics

In our sample, 49% of participants were female, and 51% were male. They were aged between 20 and 74 years, with the highest percentage found between 25 and 34 years (24%). Almost all participants (98%) were from the United States, and 80% were white. Participants were of a mixed educational background (e.g., 17% had a high school diploma as their highest level of education, 28% attended college, and 38% had a bachelor’s degree), of mixed relationship status (e.g., 51% single, 49% married or in marriage-like relationship), and of mixed income levels (spanning a yearly income between under $5,000 and over $100,000). Most participants self-identified as “middle class” (48%). Regarding their political views, most participants considered themselves to be “moderate/in the middle” (26%) or some degree of liberal (40%), while the rest stated that they were some degree of conservative (34%).

**Figure 1.**
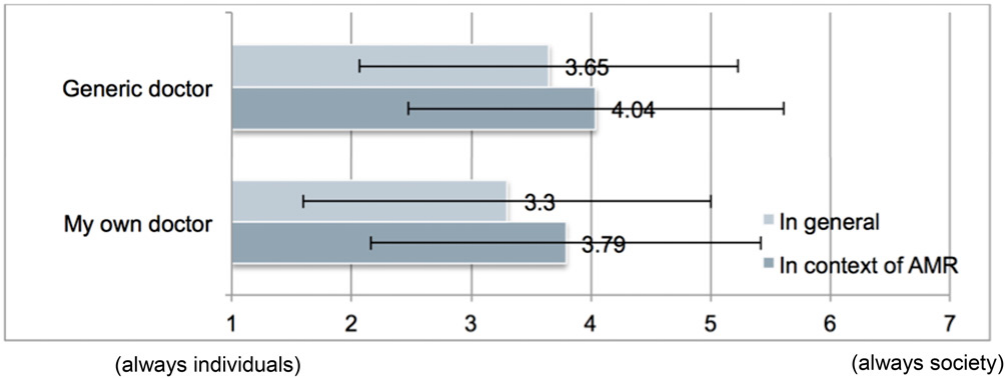
Means and standard deviations indicating prioritization of “individuals” or “society” in all four scenarios.

With regard to context, participants prioritized society significantly more when asked what a generic doctor should do (M = 3.65, SD = 1.58) compared to what their own doctor should do (M = 3.30, SD = 1.70), *t*(157) = 3.46, *p* = .001 in the general case (see Figure 1). Also for the AMR case, participants thought that generic doctors should prioritize society more (M = 4.04, SD = 1.57) compared to what their own doctor should do (M = 3.79, SD = 1.63), *t*(157) = 2.77, *p* = .006 (see Figure 1). This is perhaps not surprising: When people do not know whether they would be personally affected by a certain doctor’s behavior, as in the case of the generic doctor, it is rational for them to adopt a more impartial perspective, prioritizing society’s good over an individual’s good.

With regard to case, we found that in both contexts, participants thought that society should be prioritized more when they were informed regarding the costs of AMR and presented with a case involving an explicit risk of AMR than when asked about the general practices of doctors. This was true when participants were asked what generic doctors should do (AMR case M = 4.04, SD = 1.57; general case (M = 3.65, SD = 1.58); *t* = (157) = –2.75, *p* = .007) , and what their own doctor should do (AMR case M = 3.79, SD = 1.63; general case M = 3.30, SD = 1.70; *t*(157) = –4.00, *p* < .001).

Regarding participants’ underlying moral views, as measured by the Oxford Utilitarianism Scale, we found that the more participants subscribed to utilitarian moral views, the more they indicated society should be prioritized over individual patients in all questions reported in the preceding (all *r*s ≥ .195, all *p*s ≤ .015). The same pattern held true for both subscales, impartial beneficence (IB; all *r*s ≥ .140, all *p*s ≤ .080) and instrumental harm (IH; all *r*s ≥ .160, all *p*s ≤ .046).

For all core results, we checked for potential differences across demographic criteria. We did not find any statistically significant differences depending on demographics.

## Discussion

Which public attitudes should inform medical policy and practice? As mentioned in the preceding, ideal observer theory suggests that if public attitudes are to be given a role in determining or evidencing the ethical acceptability of medical policies and practices, we should seek the attitudes of better informed and more impartial observers. Our study suggests that those observers are likely to be more sympathetic to withholding antibiotics than the attitudes of less well-informed individuals taking a more self-interested perspective.

We found that participants were significantly more willing to deem that society should be prioritized in antibiotic prescription practices when they were made aware of AMR risks (i.e., when they were provided with knowledge of relevant facts) and when they were presented with scenarios framed in third-person terms (when taking a more impartial perspective). In our study, participants were made aware of antibiotic risks through a short educative paragraph (placed between the abstract and AMR-specific scenarios) that drew a causal link between antibiotic use and AMR development, and indicated the implications of AMR for society. We speculate that some participants had a prima facie objection to forfeiting patient welfare for the greater good, but were willing to override this objection when the social costs of antibiotic prescription were made concrete and salient.

We also found that when considering whether individual doctors should prioritize society over individual patients, participants were significantly more individualistic when they themselves were the patients and their own health was at stake than when the scenarios were presented in the third person. This was seen both in the abstract scenario and in the context of AMR.

These findings suggest that AMR health education initiatives may benefit from encouraging patients and the public to form expectations that are more impartial, and providing information about the social costs of antibiotic use in the form of AMR.

Our study showed that those who scored more highly on the OUS were more likely to prioritize society over self. This is to be expected as utilitarianism gives impartiality a central place in morality, though it is not the only theory to do so. If those members of the public who adopt the perspective of the impartial observer are best placed to have input into public policy, it could be useful to have a screening tool for identifying individuals strongly disposed to impartiality. The OUS—and IB in particular—could serve as a starting point for the development of such a tool, although it should be refined to accommodate highly impartialist nonutilitarians. Alternatively, and perhaps more controversially, if we thought that it would be more desirable to attain more balance between impartial and impartial perspective in policy processes, and assuming that a policy process already included a majority of impartial individuals, the OUS could even be used to identify those individuals who have less penchant for impartiality, so that their perspective could be taken into account during the policy process.

Our study has several limitations. As almost all participants were U.S. citizens, they would be most familiar with a fee-for-service health care system, and may have found withholding individually beneficial treatment to be less acceptable than in socialized health care systems (such as in Australia and the United Kingdom), where rationing is common. Although balanced (see demographics described earlier), our sample may not be fully representative of the U.S. population, for example. because all of our participants were required to have Internet access and to be users of the MTurk interface. Moreover, while the differences we report are statistically significant, the absolute differences on the scale used are often only small. Furthermore, the scenarios used in our survey concerned clinically mild and self-limiting infections, and we cannot generalize to more clinically severe cases.

## Conclusions

Our study provides empirical data on attitudes to antibiotic prescription of the wider public—rather than patients awaiting their consultation. It is also the first to examine the sensitivity of attitudes to awareness of AMR risks and to presentation of scenarios in terms of the first or third person, and to ask people about the trade-off between individual interests and society’s interests. We found that participants were significantly more willing to prioritize society when they were made aware of AMR risks and when they were presented with scenarios framed in the third person. We found significant associations between the OUS and prioritizing society over self, which probably reflects a greater orientation to impartiality.

Further empirical studies could be performed examining doctors’ conceptions of medical virtue and the extent to which they align their own priorities with patient welfare or public health aims. The development of a screening tool for identifying highly impartial public participants could be further explored.

## Author contributions

All authors contributed to conception, design, and drafting of the article. In addition, Bernadine Dao collected the data and wrote a first draft of the article and Nadira Faber analyzed the data.

## Conflicts of interest

None.

## Ethical approval

Ethics approval was granted from the Social Sciences & Humanities Inter-Divisional Research Ethics Committee (IDREC) of the University of Oxford on June 30, 2017 (reference number: R52226/RE001). A Confirmation of Registration was also granted from Monash University Human Research Ethics Committee (MUHREC) on July 17, 2017 (project number: 9973).

## Supplementary Material

Supplemental Material
