# Supplementary material for: Impartiality and infectious disease: Prioritizing individuals versus the collective in antibiotic prescription
Source: AJOB Empir Bioeth. 2019 Mar 25;10(1):63–9. doi: 10.1080/23294515.2019.1576799 (PMC6446247; doi:10.1080/23294515.2019.1576799)
Supplement: Supplemental Material [file UABR_A_1576799_SM3925.docx]

Appendix A: Survey

| Demographics |  |
| --- | --- |
| What year were you born in? | ______________________________ |
| What is your gender? | Female |
|  | Male |
|  | Other |
| What is your nationality? | ______________________________ |
| Which of these options best described your ethnicity? | White |
|  | Black or African American |
|  | American Indian or Alaska Native |
|  | Asian |
|  | Native Hawaiian or Pacific Islander |
|  | Hispanic or Latino |
|  | Other |
| What is your highest level of education completed? | Less than a high school degree |
|  | High School Diploma |
|  | Vocational Training |
|  | Attended College |
|  | Bachelor’s Degree |
|  | Graduate Degree |
|  | Unknown |
| What is your marital status? | Single |
|  | Married |
|  | De facto relationship |
| Please choose the category that describes the total amount of income you earned in 2017. Consider all forms of income, including salaries, tips, interest and dividend payments, scholarship support, student loans, parental support, social security, alimony, child support, and others. | Under $5,000 |
|  | $5,000-$10,000 |
|  | $10,001-$15,000 |
|  | $15,001-$25,000 |
|  | $25,001-$35,000 |
|  | $35,001-$50,000 |
|  | $50,001-$65,000 |
|  | $65,001-$80,000 |
|  | $80,001-$100,000 |
|  | Over $100,000 |
| Please indicate the option that most accurately describes your political views. | Extremely Conservative |
|  | Conservative |
|  | Slightly Conservative |
|  | Moderate/Middle |
|  | Slightly Liberal |
|  | Liberal |
|  | Extremely Liberal |
| What is the socio-economic class of the family you grew up in? | Lower class |
|  | Lower middle class |
|  | Middle class |
|  | Upper middle class |
|  | Upper class |

| Prioritization of ‘individual patients’ vs. ‘society’ in general context | |
| --- | --- |
| Doctors occasionally have to choose between decisions that are best for their patient and those that are best for society as a whole.  For example, when patients have highly infectious diseases, doctors may break patient confidentiality to notify the relevant public health authorities, and/or quarantine the patient to prevent spread of infection to others. Also, doctors may prescribe less expensive (yet less effective) drugs for their own patients, to reduce costs and allow hospitals to redistribute funds towards other patients. | |
| Do you think that in general, doctors should make decisions that prioritize their patient or society as a whole?  *Please rate on a scale of 1-7, where 1 is always prioritizing the patient and 7 is always prioritizing society as a whole.* | 1 |
|  | 2 |
|  | 3 |
|  | 4 |
|  | 5 |
|  | 6 |
|  | 7 |
| Do you think that your own doctor should make decisions about your medical care that prioritize you, or society as a whole?  *Please rate on a scale of 1-7, where 1 is always prioritizing you and 7 is always prioritizing society as a whole.* | 1 |
|  | 2 |
|  | 3 |
|  | 4 |
|  | 5 |
|  | 6 |
|  | 7 |

| Prioritization of ‘individual patients’ vs. ‘society’ in context of AMR | |
| --- | --- |
| Bacteria are a type of microbe that can be killed with drugs called ‘antibiotics’. Bacteria can become resistant to certain antibiotics – when this occurs, these antibiotics can no longer kill the bacteria. This phenomenon, called ‘antibiotic resistance’, is a type of ‘antimicrobial resistance’. Every time a patient uses antibiotics, there is a risk that ‘antimicrobial resistance’ increases in the community. This means that there are more bacteria in the community that are resistant to antibiotics. When other patients contract infections that are caused by these antibiotic-resistant bacteria, it is harder to treat their infections. This can lead to worse outcomes, and even death. | |
| Consider a scenario where a patient has a mild bacterial urinary tract infection. If the doctor did not prescribe antibiotics, the urinary tract infection would still fully resolve by itself. If the doctor did prescribe antibiotics, however, the urinary tract infection would resolve faster.  Should the doctor in this scenario prescribe the antibiotic? | Yes |
|  | No |
| When deciding whether or not to prescribe an antibiotic, doctors can prioritize their patient (by prescribing the antibiotic so that the infection heals faster), or society as a whole (by withholding the antibiotic and reducing the development of ‘antimicrobial resistance’). Do you think that doctors making decisions about antibiotic prescriptions should prioritize their patient or society as a whole?  *Please rate on a scale of 1-7, where 1 is always prioritizing the patient and 7 is always prioritizing society as a whole.* | 1 |
|  | 2 |
|  | 3 |
|  | 4 |
|  | 5 |
|  | 6 |
|  | 7 |
| There are some situations where your own health would improve with antibiotic treatment – for example, if you had a mild bacterial infection. This is a test question to make sure you are paying attention.  *Please skip this question and leave the answer blank.* | 1 |
|  | 2 |
|  | 3 |
|  | 4 |
|  | 5 |
|  | 6 |
|  | 7 |
| There are some situations where your own health would improve with antibiotic treatment – for example, if you had a mild bacterial infection. Do you think that in general, your own doctor should make decisions about prescribing antibiotics to you, which prioritize your own health (by prescribing the antibiotic) or that of society as a whole (by withholding antibiotic treatment)?  *Please rate on a scale of 1-7, where 1 is always prioritizing you and 7 is always prioritizing society as a whole.* | 1 |
|  | 2 |
|  | 3 |
|  | 4 |
|  | 5 |
|  | 6 |
|  | 7 |
